# Functional Electrical Stimulation of Peroneal Muscles on Balance in Healthy Females

**DOI:** 10.34133/2021/9801097

**Published:** 2021-05-13

**Authors:** Zoe A. Bamber, Wei Sun, Rhea S. Menon, Patrick C. Wheeler, Ian D. Swain, Daniel T. P. Fong

**Affiliations:** ^1^National Centre for Sport and Exercise Medicine, School of Sport, Exercise and Health Sciences, Loughborough University, Loughborough, UK; ^2^Division of Orthopaedics, Trauma and Sports Medicine, School of Medicine, Faculty of Medicine and Health Sciences, University of Nottingham, Nottingham, UK; ^3^Shandong Sport University, Jinan, Shandong, China; ^4^Department of Sport and Exercise Medicine, University Hospitals of Leicester NHS Trust, Leicester, UK; ^5^The Faculty of Science and Technology, Bournemouth University, Poole, Dorset, UK

## Abstract

Balance improvement could contribute to ankle stability for the prevention of ankle sprains. Functional electrical stimulation (FES) is an effective way of augmenting muscle activity and improving balance. This study investigated the effect of FES of peroneal muscles on single-and double-leg balance. Fifteen healthy females (age = 23.1 ± 1.6 years, height = 1.63 ± 0.07 m, and weight = 63.7 ± 9.9 kg) performed single- and double-leg standing balance tests with eyes open and closed before and after 15-minute FES intervention during treadmill running at a comfortable, self-selected pace. FES of peroneal muscles was provided bilaterally, using an Odstock Dropped Foot Stimulator. The total excursion of the centre of pressure (COP) was calculated to assess the standing balance control ability. The total excursion of COP in single- and double-leg stance with eyes open reduced significantly after FES intervention by 14.7% (*p* < 0.001) and 5.9% (*p* = 0.031), respectively. The eyes-closed condition exhibited a 12.7% (*p* = 0.002) reduction in single-leg stance but did not significantly change in double-leg stance (*p* > 0.05). Limb preference did not account for balance postintervention. No significant difference in total excursion of COP was found between preferred and less preferred limbs with both visual conditions (*p* > 0.05). FES of peroneal muscles improved standing balance control with eyes open in double-leg and single-leg stance and with eyes closed in double-leg stance. The improvements in balance control with FES treatment did not vary concerning limb preference.

## 1. Introduction

Ankle sprains are one of the most common musculoskeletal injuries accounting for about 23% and 15% of injuries sustained during high school and college education, respectively [1, 2]. People suffering from repeated ankle sprains are more likely to develop chronic ankle instability [3], which may lead to less flexible movement patterns [4], thus a tendency to minimise the reliance on ankle movement in walking and running [5] and higher visual reliance [6] and more postural sway in balancing task [7, 8]. The recurrence rate for a lateral ankle sprain is reportedly as high as 80% among athletes, responsible for the longest absenteeism from participation compared to other sports injuries [2]. Ankle injuries also have a drastic impact on the health care system, with an estimation of 1-1.5 million people in the United Kingdom attending emergency rooms and clinics yearly, with medical costs amounting to £1-£2 billion annually [9]. Female athletes suffer ankle sprain more often than male counterparts for sex-specific differences in ankle stiffness, degrees of freedom, and muscle strength [10]. Therefore, it is important to prevent the incidence or recurrence of ankle injuries, especially among females.

Postural control stability deficits are frequently reported following an acute ankle sprain [11]. It has been suggested that impaired postural control during spontaneous dynamic activity increases the risk of noncontact inversion ankle sprain [12]. Standing balance ability is a characteristic of postural control with an assessment of the centre of pressure (COP), which is the location of the vertical projection of the ground reaction force measured with a force platform [13]. Studies have demonstrated good test-retest reliability for COP parameters including total excursion, sway velocity, and sway area during balance testing for various measurement conditions [14]. Total excursion (TE) of COP is the total trajectory of the COP from its start position to the maximal position. Lower total excursion is indicative of better balance [15]. Several physical therapy applications including bracing [16], Kinesio taping [17], Tai Chi exercises [13], and balance training [18] have been used for improving standing balance ability. However, the immediate effects of physical therapy were usually not significant, and prompt effective therapy options are needed to further design long-term training programs to achieve significant improvement in balance.

Functional electrical stimulation (FES) is an effective way to activate muscle activity in which the electrical stimulation was triggered with a foot stimulator [19]. Peroneal muscles have been shown to play an important role in balance control ability during difficult standing tasks [20]. Peroneal muscles contribute largely to lateral ankle stability and it has been implied that training these muscles could help prevent ankle sprains [21–24]. Decreased evertor activation during functional activities has been reported in participants with inferior ankle stability [25]. On the contrary, a previous study demonstrated that increased leg muscle activity contributes to greater postural instability [26]. They contemplate that training peroneus muscles might be an inefficient training strategy to improve balance, since this may lead to larger compensations in medial-lateral sway and instead compromise balance.

Although the evidence is controversial, electrical stimulation has been used vastly as a treatment to improve swelling and pain following acute ankle sprains [27]. However, whether FES treatment can be used to efficiently train the lateral peroneal muscles to improve ankle stability and possibly prevent ankle sprain injury in the process of doing so is still unknown. The current study is aimed at observing the short-term, immediate effects of FES of the peroneal muscles to investigate whether it can be used for prevention of ankle sprains in the future. Therefore, this study assessed the effect of FES of peroneal muscles on standing balance ability. It is hypothesised that single-leg as well as the double-leg balance with both eyes-open and eyes-closed conditions will improve following immediate FES of lateral peroneal muscles.

## 2. Methods

### 2.1. Participants

Sample size estimation was done in G∗Power software (Germany), based on a previous study, which reported that the total excursion of COP reduced from 802.61 ± 77.72 mm to 750.14 ± 71.03 mm with eyes open in double-leg stance after balance training [28]. By setting the level of significance to 0.05 and the statistical power to 0.80 in a one-tailed test on matched pairs, the effect size was calculated to be 0.703, and the estimated required sample size was calculated to be 14. Fifteen healthy, recreationally active females (age = 23.1 ± 1.6 years, height = 1.63 ± 0.07 m, weight = 63.7 ± 9.9 kg, and BMI = 24.0 ± 3.5 kg/m^2^) were recruited with invitation letters, advertisements, and personal invitations. With sample size of 15, setting the level of significance to 0.05, in a one-tailed test, the power of all statistical comparisons in this study ranged from 0.801 to 0.852. Exclusion criteria consisted of any conditions which could interfere with normal stability: lower extremity injury (<6 months), balance disorders (e.g., vertigo and vestibular dysfunction), medical pathologies disturbing balance, sensory impairments, comorbidities such as diabetes mellitus and hypertension, cardiac conditions (e.g., pacemaker), and pregnancy as an electric current was involved in the intervention. The study was approved by the Loughborough University Research Ethics Committee (R18-P070). All the participants gave their written informed consent.

### 2.2. Experimental Procedure

**Figure 1:**
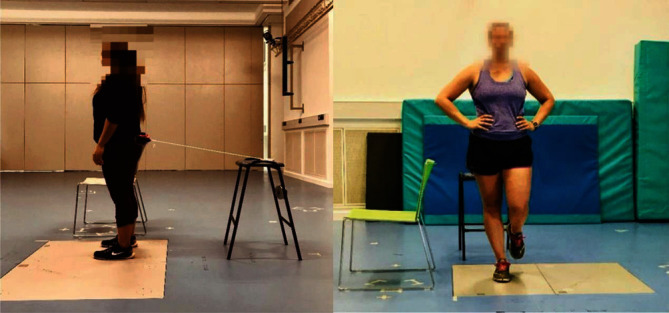
Experimental setup of the (a) double-leg and (b) single-leg stance balance test with the custom-made perturbation system.

Single- and double-leg stance balance tests were conducted with a force plate (Kistler 9287C, 900 × 600 mm, Switzerland) at 1000 Hz before and immediately after FES intervention in a quiet room. During single-leg stance balance tests, the participants were instructed to stand on one leg on the force plate, wearing their footwear as motionless as possible, respectively (Figure 1). The other leg was flexed to 90 degrees at the knee joint, and both arms were placed on the sides of the waist. A total of 10 seconds' balance test data was collected during a single-leg stance with eyes open and closed when the participants have started to maintain balance. The trial failed if the supporting leg moved or the nonsupporting leg touched the plate. Both legs were tested in turn, with the preferred leg being defined by the participant as the leg they preferred to use for kicking a football [29]. During double-leg stance balance tests, again the participants stood on the force plate wearing their footwear, feet shoulder-width apart, and both arms by their sides for 30 seconds with eyes open and closed. For the double-leg stance test, external posterior perturbations were provided using a custom-made perturbation system (Figure 1) in the eyes-open condition to make it more challenging and simulate realistic balance threats [14]. This system included an inelastic belt worn at the waist, attached to a 1-meter rope used as a cable, which in turn was tied to weight plates.

The rope was passed over a stool of 3 feet (0.914 meters) in height, where the weight plates were placed. As the height of the centre of gravity was suggested to be at 56% of the body height in the literature [30], this 3-foot tall stool was the height of the centre of gravity of female of 1.63 meters tall, and this is equally exactly the mean height of the participants in this study. The range of the height of the participants was from 1.49 to 1.73 meters, with the greatest vertical difference of 0.078 meters between the height of the centre of gravity and that of the stool. With this difference and the length of the rope being 1 meter long, the horizontal component of the perturbation force was at least 99.7% of that of a pure horizontal pull. As the loss of the horizontal pulling force was small as it was within 0.3% only, we kept the height of the stool throughout the entire experiment without adjusting it for each participant.

The cable pulls were applied by dropping the weight plates at random instances during the data collection, to provide the posterior perturbation via a pulley-like effect. Weight dropped was calculated at 10% of body weight [31]. Three successful trials were tested following one practice trial for each one of six conditions (single-leg stance for right or left leg, double-leg stance, with eyes open and closed) with random orders. The assessment was done 1 min after the intervention. The time interval for breaks was 1 min between two consecutive trials.

### 2.3. Data Analysis

COP data was extracted using BioWare software (Version 5.1.3.0, Switzerland) and filtered with a low pass Butterworth filter at 40 Hz [32]. Total excursion was calculated with the total distance COP travelled between the two successive time points, which were 1/1000 second apart. Distance between successive $\text{time points}=\sum_{i=1}^{N-1}\sqrt{{\left(X_{i+1}-X_{i}\right)}^{2}+{\left(Y_{i+1}-Y_{i}\right)}^{2}}$, where *X* and *Y* are COP coordinate values [33]. A single value for COP total excursion was obtained and analysed for each task. Total excursion values were also averaged combining the single preferred and less preferred limb values to determine the effect on the single-leg stance as a whole. Percentage change in mean scores from pre- to postintervention was calculated.

Several similar COP-related data, such as average sway velocity, anterior-posterior velocity, medial-lateral velocity, and total excursion area, have been used in previous studies [34]; however, the total excursion (or trajectory) length has been suggested to be a complementary measure to maximum excursion to better reflect balance in multiple directions [15]. As using too many variables would reduce the power of a study, we decided to analyse only the total excursion length in this study.

### 2.4. Intervention

**Figure 2:**
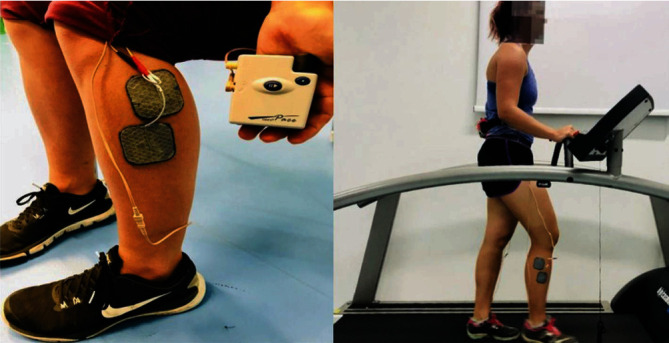
(a) Placement of the active/inactive electrode on the upper lateral shank, and (b) treadmill running with the FES device equipped bilaterally.

FES to the peroneal muscles was delivered by using the Odstock Dropped Foot Stimulator (ODFS®-Pace V1.0, Odstock Medical Limited, UK). The pocket-sized digital device provides stimulation via self-adhesive surface electrodes. Output amplitude for the participants was adjusted until a visible stimulated contraction was achieved and ranged from 20 mA to 45 mA (pulse width 180 *μ*s, frequency 40 Hz) [35]. The active electrode is placed near the head of the fibula, with the inactive electrode placed below and slightly forwards (Figure 2), following the SENIAM recommendation [36] and a previous study [24]. A footswitch is placed under the heel of the foot which allows for stimulation to be triggered when weight is placed on (heel strike) the footswitch. The footswitch enables a particular muscle to be stimulated at the correct time in the gait cycle. The stimulation comes on when the heel switch is activated on the heel strike to cause ankle eversion. The participants received 15 minutes [19] of FES to the peroneal muscles bilaterally, during treadmill running at a comfortable pace. The intervention was running as this can be repeatedly done on a treadmill for 15 minutes. The muscle stimulation was delivered at heel strike as ankle sprains happened mainly within 50 ms after heel strike and the peroneal muscles only started to work 50 ms after the heel strike [37], so the balance and stability of the ankle joint are more important during the initial phase right after the heel strike.

### 2.5. Statistical Analysis

The software SPSS 20.0 (IBM, New York, USA) was used for data analysis, and all the parameters were presented as mean ± standard deviation. The different task conditions (double-leg stance, single-leg stance, eyes open, and eyes closed), time (preintervention, postintervention), and limbs (preferred limb, less preferred limb) are independent variables. All parameters showed normality with the Shapiro-Wilk test. A two-way analysis of variance (time × limb) was done to see any significant effects by time and limb. Paired *t*-tests were used to compare preintervention and postintervention total excursion of COP for each task condition. Statistical significance was set at *p* < 0.05.

## 3. Results

Table 1 shows the total excursion distance in the preintervention and postintervention tests in all tested conditions. The repeated measures ANOVA demonstrates a significant effect of FES intervention (Wilks' lambda = 0.008, *F* = 43.35, *p* = 0.001) across the different tested conditions.

### 3.1. Total Excursion in Double-Leg Stance, with Posterior Perturbations

Total excursion in double-leg stance with eyes open decreased significantly by 5.9% (7.465 ± 1.529 m vs. 7.052 ± 1.796 m, *t* = 2.402, *p* = 0.031) after intervention. The total excursion in double-leg stance with eyes closed decreased by 3.65% (7.851 ± 1.952 m vs. 7.578 ± 1.326 m, *t* = 1.159, *p* = 0.266) after intervention.

### 3.2. Total Excursion in Single-Leg Stance (Combined Preferred and Less Preferred Leg)

Total excursion in single-leg stance (combined preferred and less preferred leg) with eyes open decreased significantly by 14.7% (2.971 ± 0.755 m vs. 2.591 ± 0.708 m, *t* = 3.972, *p* < 0.001) after intervention. Total excursion in single-leg stance (combined preferred and less preferred leg) with eyes closed decreased significantly by 12.7% (3.210 ± 0.754 m vs. 2.848 ± 0.67 m, *t* = 3.372, *p* = 0.002) after intervention.

### 3.3. Total Excursion in Single-Leg Stance (Preferred Leg)

Total excursion in single-leg stance (preferred leg) with eyes open decreased significantly by 21.7% (3.004 ± 0.412 m vs. 2.468 ± 0.582 m, *t* = 5.820, *p* < 0.001) after intervention. Total excursion in single-leg stance (preferred leg) with eyes closed decreased significantly by 13.9% (3.189 ± 0.823 m vs. 2.801 ± 0.611 m, *t* = 2.670, *p* = 0.018) after intervention.

### 3.4. Total Excursion in Single-Leg Stance (Less Preferred Leg)

Total excursion in single-leg stance (less preferred leg) with eyes open decreased significantly by 16.7% (2.829 ± 0.793 m vs. 2.424 ± 0.804 m, *t* = 3.311, *p* = 0.005) after intervention. Total excursion in single-leg stance (less preferred leg) with eyes closed decreased significantly by 11.6% (3.231 ± 0.708 m vs. 2.896 ± 0.743 m, *t* = 2.061, *p* = 0.058) after intervention.

## 4. Discussion

In the current study, the effect of FES of peroneal muscles on balance in double-leg as well as single-leg stance was assessed with both visual conditions. The main findings were the significant difference in total excursion values between pre- and postintervention for the double-leg stance with eyes open and the single-leg stance with eyes open as well as eyes closed. In the conditions, the reduction in total excursion values of COP after FES treatment indicated the improvement in balance control ability in this study. This is in line with some previous findings [38, 39] which showed significant balance improvements after using electrical stimulation. Two factors might lead to this phenomenon. On the one hand, muscle activity around the ankle joint plays an important role in maintaining balance and postural stability. High correlations were reported between balance control during arduous tasks and peroneal muscle activity, and there was a greater decrease in sway with an increase in peroneus longus EMG activity [26]. It is inferred that the higher level of activity is required in the peroneal muscles when the demand for stability increased, which is precisely what FES aims to do. FES training could activate the skeletal muscle, facilitate ankle stabilising muscle contraction, and enhance neuromuscular control around the ankle [40]. On the other hand, reaction time was defined as the time between ankle perturbation and the onset of peroneal muscle activity [41]. The patients with chronic ankle instability had been reported a delayed reaction time of peroneal muscles [42] and postural control deficits represented by increasing total excursion and area of the COP [43]. At neuromuscular, cellular, and molecular levels, FES training could significantly increase the fast-type myofibres' diameter of trained muscles and reduce the premotor reaction time by 18% [44]. It is crucial to note that a distinctive feature of electrical stimulation is the nonselective recruitment of fibres [40]. The ability to recruit fast muscle fibres that are not normally recruited during most daily activities provides added gains in muscle performance, another plausible explanation for the improvement in balance in our study. This could potentially play a more significant role in individuals who need to improve balance postinjury, as the healing effect of artificially activating these fibres is believed to help mitigate the responses to disuse and accelerate recovery [40]. The shortened reaction time might be another factor to improve balance after FES of peroneal muscles.

It is noteworthy that there was no significant difference in total excursion found in double leg and less preferred leg with eyes closed between pre- and post-FES intervention in this study. Vision is one of the most important information input system contributing to standing balance ability; the trunk sway increased threefold without visual condition in standing balance test [45]. The lack of vision could be the probable reason why the balance did not improve for the eyes-closed condition in this study. The balance control for eyes closed might mostly rely on proprioception in lower limb joints; the proprioceptive input information plays an essential role in postural control and integration of motor control [46]. For the eyes-closed condition, as the demand for stability is higher, the proprioceptive requirement is greater. The present study might speculate that the FES (range from 20 mA to 45 mA, pulse width 180 *μ*s, and frequency 40 Hz) to the peroneal muscles could not improve proprioception enough to increase standing balance without visual condition; potentially more stimulus is required to produce the desired effect.

This is following the findings reported by studies that demonstrated that lower-limb dominance did not influence single-leg balance among healthy young adults [47]. An underlying reason could be that, as the stimulation was delivered in response to heel strike on the footswitch, the same output of FES which was delivered to both limbs was identical, irrespective of limb preference, and thereby curtailed any possible variation in treatment. It can be seen from this that the improvement of standing balance control with a single leg could be symmetrical after FES intervention, which is particularly important for preventing ankle sprain when clinicians and researchers treat ankle injuries with FES.

The potential application of findings is valuable for improving the standing balance in chronic ankle instability. This study showed that FES could improve the standing balance ability in healthy adults, which might be used for chronic ankle instability. However, the results of this study are from healthy adults, with no chronic ankle instability, and should be applied with caution. Further study could focus on the effects of FES on the balance in chronic ankle instability.

This work has four limitations. Firstly, this is a laboratory-based cohort study so there was not a control group. We could only demonstrate the overall effect after the intervention, but cannot demonstrate if the effect is from the muscle stimulation or the running exercise. However, the results from this study are essential for demonstrating the feasibility of the intervention, before a future randomised controlled study with a control group is planned. Secondly, we recruited only healthy young female adults in this study, as the purpose was to demonstrate if the intervention is feasible. We suggested that future studies should consider other populations such as people with chronic ankle instability. Thirdly, we only demonstrated the immediate effects right after the intervention. We suggested that future studies can further investigate the effect after 30-60 minutes of the intervention and, if time allows, days or weeks after the intervention to demonstrate the longer-term effect. Finally, we measured only the total excursion distance as to avoid losing the power of the study, but we suggested that future larger-size clinical trials should consider adding some functional performance parameters to show the overall clinical effect of the intervention.

## 5. Conclusion

Immediate FES of the peroneal muscles proves to be effective in improving standing balance control with eyes open in both double-leg and single-leg stance. Long-term follow-up FES is required for confirming the effects on balance control with eyes open and closed. The improvements in balance control with FES treatment do not vary concerning limb preference. The novel aspect of the current study is that the stimulation was done functionally but not just as a stand-alone acute treatment. It is interesting to mention that a recent systematic review [48] found that postural balance can be improved by training the strength of the toe flexor, though in older people over the age of 60 only. Future studies can investigate the effect of FES on both peroneal muscles and toe flexor for improving balance in younger adults.

## Figures and Tables

**Table 1:** Total excursion distance (in meters) in the preintervention and postintervention tests in all tested conditions.

	Preintervention	Postintervention	Percentage change	*t*	*p* value
Double leg, eyes open, with posterior perturbations, 30 s	7.465 ± 1.529	7.052 ± 1.796	-5.9%	2.402	0.031^∗^
Double leg, eyes closed, with posterior perturbations, 30 s	7.851 ± 1.952	7.578 ± 1.326	-3.65	1.159	0.266
Single leg (combined preferred and less preferred), eyes open, 10 s	2.971 ± 0.755	2.591 ± 0.708	-14.7%	3.972	<0.001^∗^
Single leg (combined preferred and less preferred), eyes closed, 10 s	3.210 ± 0.754	2.848 ± 0.670	-12.7%	3.372	0.002^∗^
Single leg (preferred), eyes open, 10 s	3.004 ± 0.412	2.468 ± 0.582	-21.7%	5.820	<0.001^∗^
Single leg (preferred), eyes closed, 10 s	3.189 ± 0.823	2.801 ± 0.611	-13.9%	2.670	0.018^∗^
Single leg (less preferred), eyes open, 10 s	2.829 ± 0.793	2.424 ± 0.804	-16.7%	3.311	0.005^∗^
Single leg (less preferred), eyes closed, 10 s	3.231 ± 0.708	2.896 ± 0.743	-11.6%	2.061	0.058

^∗^ *p* < 0.05.

## Data Availability

The data used to support the findings of this study are available from the corresponding author upon request.
